# Peer interventions to improve HIV testing uptake among immigrants: A realist review

**DOI:** 10.34172/hpp.42639

**Published:** 2024-03-14

**Authors:** Elham Ghasemi, Tahereh Bahrami, Reza Majdzadeh, Reza Negarandeh, Fatemeh Rajabi

**Affiliations:** ^1^Community Based Participatory Research Center, Tehran University of Medical Sciences, Tehran, Iran; ^2^Medical Ethics and Law Research Center, Shahid Beheshti University of Medical Sciences, Tehran, Iran; ^3^School of Health and Social Care, University of Essex, Colchester, UK; ^4^Knowledge Utilization Research Center, Tehran University of Medical Sciences, Tehran, Iran; ^5^Nursing and Midwifery Care Research Center, School of Nursing and Midwifery, Tehran University of Medical Sciences, Tehran, Iran; ^6^Center for Academic and Health Policy, Tehran University of Medical Sciences, Tehran, Iran

**Keywords:** Acquired immunodeficiency syndrome, Emigrants and immigrants, HIV, HIV testing, Realist review, Refugees

## Abstract

**Background:**

As a vulnerable group in HIV control programs, immigrants face various obstacles to HIV testing. Despite the effectiveness of peer interventions on health promotion in HIV testing, relatively little is known about how these interventions work. This realist review aims to understand why, how, and under what conditions peer interventions can improve immigrants’ HIV testing uptake.

**Methods:**

We followed the steps suggested by Pawson and colleagues for conducting the realist review. To test a initial program theory, we first systematically searched databases of PubMed, Web of Science, Scopus, Embase, and Cochrane, as well as the websites of UNAIDS, World Bank, Global Fund, WHO, and IOM. After data extraction and quality appraisal, data synthesis was conducted to explain the intervention pathways corresponding to context-mechanism-outcome configurations.

**Results:**

Seventeen studies were included in the review. Peer interventions for improving immigrants’ HIV testing uptake worked through four pathways: Following the improvement of communications (as a proximal mechanism): 1) increasing awareness, 2) reduced stigma, 3) improved support, and 4) increased access to services could lead to improved HIV testing uptake among immigrants. The identified mechanisms were influenced by three groups of individual/ interpersonal, service delivery, and structural factors.

**Conclusion:**

Peer interventions with multiple strategies to be designed and implemented considering the barriers to HIV testing and also moving beyond one-size-fits-all approaches can successfully improve the immigrants’ HIV testing uptake. The refined program theory in this study can help the healthcare providers and policy-makers promote the immigrants’ HIV testing uptake and reduce the risk of disease transmission.

## Introduction

 Human immunodeficiency virus (HIV) continues to be a major global public health issue. In 2022, 1.3 million new HIV cases were identified worldwide, where 630 000 people have died of acquired immune deficiency syndrome (AIDS)-related illnesses. The total number of people living with HIV was estimated at 39 million worldwide.^1^ HIV testing and diagnosis are regarded as the key components of the 95-95-95 testing and treatment targets to end the AIDS epidemic. The effective implementation of HIV testing services has been introduced as a crucial component of success in responding to HIV control within national and global measures.^2^

 Population mobility is recognized as a driver of the HIV epidemic^3,4^ by linking geographically separate epidemics and intensifying transmission by inducing riskier sexual behaviors.^3^ Immigrants are considered a mobile sub-population-group in HIV targets for 2025.^5^ Complex vulnerabilities can overlap to limit immigrants’ access to existing services and put them at heightened risk of HIV, including irregular immigration status, the experience of stigma, discrimination and marginalization, poverty and lack of employment opportunities, legal barriers and lack of legal protections, language and cultural barriers.^6^ Despite the existence of high-risk HIV behaviors in immigrants and ethnic minorities,^7,8^ there is evidence that immigrants face various barriers to accessing HIV testing services at individual, social, and structural levels,^9-11^ and the multiplicity of these barriers indicates the complexity of the response.

 According to the World Health Organization (WHO) consolidated guidelines on HIV testing services, demand creation to increase HIV testing service uptake and engage those in greatest need of services was introduced as a valuable tool for mitigating stigma, discrimination, and criminalization. These strategies include activities intended to improve an individual’s knowledge, attitudes, motivations, and intentions to test and inform the decision to obtain HIV testing services. In this area, peer-led demand creation interventions have been recommended to create the demand for HIV testing.^2^ Similar to the target group in various aspects of their social identities, Peers can provide support in ways that non-peer professionals are less likely to accomplish. In addition, peers who constantly interact with the members of their social network have better access to hard-to-reach populations.^12^ The peer education model is regarded as an effective approach to raising awareness and also providing better access to HIV/AIDS services for hard-to-reach populations,^13,14^ reducing HIV infection, as well as improving HIV testing.^15^ HIV peer interventions have been identified as low-cost^15^ and cost-effective^16^ interventions, and it is widely recommended in areas with limited resources.^15^

 Despite the effectiveness of peer interventions on health outcomes in different aspects of HIV prevention and control in the immigrant population,^17-20^ however, relatively little is known about how these interventions work, for whom, and in what circumstances. In the methodological evidence for complex interventions, identifying the mechanisms that produce outcomes and the contexts that influence their implementation are emphasized.^21,22^ In general, individuals might not have similar choices about their behavior, and such decisions, such as HIV testing, are mainly dependent on the opportunities and resources provided by the interventions.^23^ In this regard, by developing Context-Mechanism-Outcome Configurations (CMOCs) during the process of analysis and generating program theory, a realist review can identify how an intervention or action under certain contextual circumstances (C) may trigger a mechanism (M) to achieve a given outcome (O).^24,25^ Realist review is a type of systematic literature review with a explanatory focus.^25^ In a realist review, the first step is to make explicit a program theory.^24^ The term ‘program theory’ refers to “an abstracted description and/or diagram that lays out what a program (or family of programs or intervention) comprises and how it is expected to work”.^25^ Empirical evidence is then sought to populate this theoretical framework, supporting, contradicting, or modifying the program theory.^24^ It can help policymakers and health managers assess whether effective interventions in one setting may work so in another and tailor the interventions for specific contexts.^24^ Considering the importance of HIV prevention and control in international immigrants,^26^ this realist review was conducted to address why, how, and under what conditions peer interventions can improve HIV testing uptake among this population.

## Material and Methods

 The five steps guided us in conducting a realist review outlined by Pawson et al^24^ and reported following the Realist And MEta-narrative Evidence Syntheses: Evolving Standards (RAMESES) publication standards.^25^

###  Clarifying the scope of the research

 Our research question was proposed to determine the scope of the research: “How and under what circumstances do peer intervention strategies influence HIV test uptake among immigrants?”.

**Figure 1:**
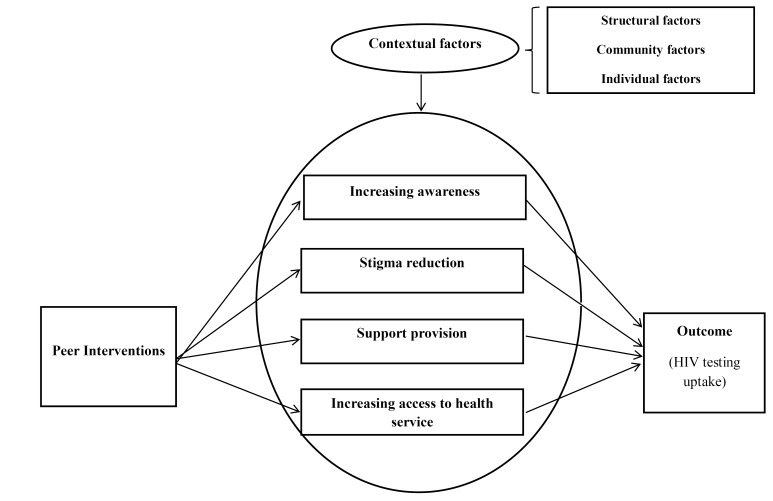
The initial program theory explaining the effect of peer interventions on immigrants’ HIV testing uptake.

 A program theory is usually a diagram describing an intervention’s activities, the intended outcomes, and the mechanisms that lead to the outcomes.^25^ To generate the initial program theory, the review team first prepared an initial draft of the framework explaining the effect of peer interventions on immigrants’ HIV testing uptake based on their knowledge of health promotion interventions, improving access to HIV services, and immigrant health. Then, it was modified through interviews with stakeholders regarding strategies to improve access to HIV testing services among immigrants and a review of the related literature. Finally, the initial program theory was designed to test (Figure 1). The main concepts of the initial program theory are presented in Table 1.

**Table 1:** The description of the concepts of the initial program theory

**The concept **	**Description**
Peer intervention	Any kind of educational, support, and consulting intervention or providing a particular service to an immigrant or a group of immigrants by a peer or a group of peers (with common demographic characteristics and/or experiences).
Increasing awareness	The promotion of HIV knowledge through receiving formal and informal information.
Reducing stigma	Reduce the perception or experience of HIV-related stigma as well as other levels of stigma (related to a particular social identity, such as race, gender, migration, and/or sexual orientation)
Support	The provision of resources and communication with others that can facilitate dealing with the problem, including emotional support (concern, acceptance, perception, encouragement, and trust), financial support (financial aid and related services), and/or information support (knowledge, recommendation, and feedback).
Increasing access to the services	Improvement in access dimensions, such as geographical access to services, as well as availability, affordability, and acceptability of services
Contextual factors	Factors in levels of Individual (age, gender, race, social class, occupation, etc), community (culture, stigma, etc.), and/or structural (regulations, policies, organizational structures, etc).
Outcomes	Using HIV testing services (self-test, rapid test, serology, or antibody testing ) through CITC or PITC approaches.

Abbreviations: CITC, client-initiated testing and counseling; PITC, provider-initiated testing and counseling.

###  Searching for evidence and selecting studies

 The search resources included the following electronic databases: PubMed, Web of Science, Scopus, Embase, and Cochrane; the websites of related international organizations: The Joint United Nations Programmes on HIV/AIDS (UNAIDS), the World Bank, Global Fund, WHO, and International Organization for Migration (IOM); as well as the reference list of the related studies. The keywords were obtained from various resources such as related articles and experts’ opinions.

###  Inclusion criteria

 For a realistic review, several primary studies (interventional or non-interventional) were taken into account with a variety of designs, provided that these studies have targeted the international immigrants or refugees population. Interventional studies (based on RCT or non-RCT design) should be conducted with any educational, supportive, or counseling interventions performed by an individual or a group of peers, and HIV testing should be considered as one of the intervention outcomes. In the case of non-interventional studies, the researchers selected empirical or conceptual/theoretical studies with a variety of designs as well as quantitative, qualitative, and/or mixed methods that describe or explain the concepts and relationships in the initial program theory. In other words, the selected studies should include data on how an intervention or service under certain contextual circumstances may trigger a mechanism to improve HIV testing uptake. Given that new HIV testing for the public was first approved in 1985,^27^ the search period was considered from the beginning of 1985.

###  Exclusion criteria

 Secondary studies or studies that were published in a non-English language; studies with the target populations such as domestic immigrants, non-immigrant ethnic/racial minorities, or indigenous populations; no report on HIV testing as the study outcome, and the studies whose findings were not relevant to confirm, refute, or refine initial theory.

 Studies were reviewed in accordance with the Preferred Reporting Items for Systematic Reviews and Meta-Analyses (PRISMA) guidelines.^28^First, the abstracts were screened based on the study selection criteria. Then, two reviewers independently evaluated the full text of the articles to select relevant studies. In other words, studies were selected based on whether they could inform the development of the initial program theory or clarify the CMOCs.^24^ Disagreements between the two reviewers were resolved through consensus.

###  Quality appraisal

 In a realist review, the evaluation of the quality of the included studies is mainly based on the researchers’ judgments about the relevance and rigor criteria. For relevance, we should assess whether the study addresses the theory under test. Rigor also refers to whether a certain investigator’s inference has sufficient weight to provide a valid methodological contribution to the testing of the intervention theory.^24,25^ The present study selected relevant articles containing data regarding the initial program theory. In other words, these articles would be judged to determine whether the content of a part of the study contained the data pertinent to the formation of the program theory. In addition, and according to each study type, the Joanna Briggs Institute (JBI)’s Critical Appraisal Tools^29^ were used for quality appraisal. Eventually, the quality of selected studies was evaluated based on the relationship between studies data in explaining the program theory, the number of CMOCs extracted from studies, as well as the results of the assessments by JBI tools. In the present study, this step was also performed by two reviewers independently. In case of any disagreement, they would discuss the issue to reach an agreement.

###  Data extraction

 The data extraction form included items pertaining to the study characteristics, strategies, and theory concepts, such as contextual factors, mechanism, and outcome. Then, the data were extracted from three studies using different designs to finalize this form. The two reviewers independently completed the ultimate data extraction form for the selected studies. Then, the respective results were compared by the two reviewers, and agreement was also reached over contradictory issues.

###  Data analysis, synthesis, and conclusion

 The thematic analysis method was used to analyze the data. At first, each study was reviewed several times, and the meaning units of data extracted from each study were identified in terms of intervention strategies, contextual factors, and mechanisms. In the next step, the meaning units were coded. The codes were compared in terms of similarities and differences. These were then reviewed, compared, and grouped to determine the categories. Similar categories were merged, and themes were extracted.

 Furthermore, CMOCs were identified and validated by comparing the mechanisms in CMOCs in similar and different contexts to summarize the nature of CMOCs relationships. The pathways were identified and verified accordingly. Finally, the initial program theory was refined to reflect the evidence-supported mechanisms. All interpretive processes were discussed and agreed upon among the review’s contributors.

## Results

**Figure 2:**
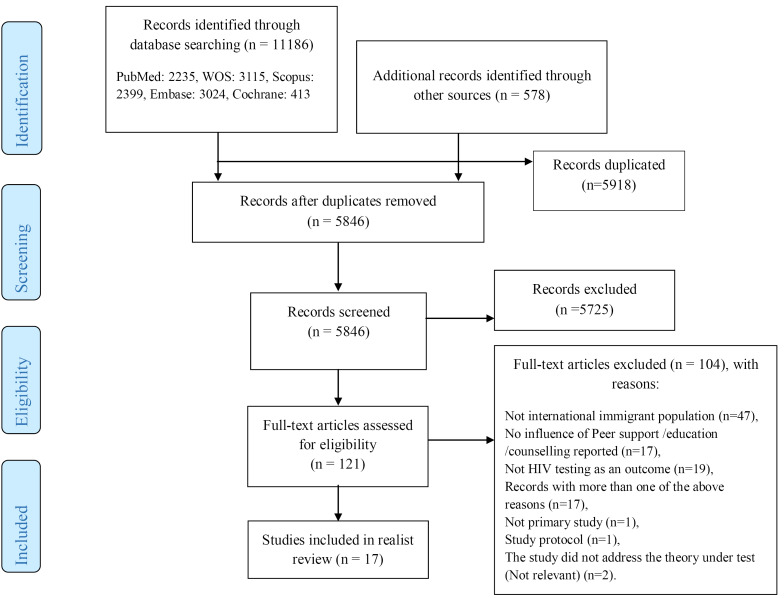
PRISMA flowchart for study selection

 The selection process for the studies is presented in PRISMA flowchart (Figure 2). It should be noted that a total of 5846 study titles and abstracts were reviewed; consequently, 17 studies were eligible to enter the present realist review.

###  Study characteristics

 Ten studies used a quantitative approach (with various RCT, quasi-experimental, cross-sectional, and cohort designs), and 7 studies used a qualitative approach. In studies with the interventional design (n = 6), the effectiveness of interventions on the outcome of HIV testing was reported. In terms of providing intervention or service, 10 studies were regarded as interventional studies, while no intervention or service was provided in the other 7 studies. The majority of studies had focused on key populations for HIV, including sex workers, men who have sex with men (MSM), and gay, bisexual and other men who have sex with men (GBMSM) immigrants. Most studies were conducted on the Latin immigrants population in the United States (n = 10), with one of these along the US-Mexico border. The remaining seven studies were conducted in the Mexico-Guatemala borderline (n = 2), Asia (n = 2), Europe (n = 1), Oceania (n = 1), and Africa (n = 1). Further information regarding the included studies is provided in Table 2.

**Table 2:** Summary of studies in realist review

**Author** **(Date)**	**Country**	**Intervention/Service**	**Intervention Strategies**	**Study type**	**Target Population**	**Key findings**	**QA scores**
Febres-Cordero et al,^36^ 2020	Mexico- Guatemala border	-	-	Qualitative	Migrant women sex workers	Primary sources of information related to HIV/ STI prevention, sexual and reproductive health, and safety included other sex workers; managers within certain (supportive) indoor establishments; doctors; health outreach workers; and—to a lesser extent—family, friends, and media. Most accessed or shared such information face-to-face or via phone or text messages. Variations in access and types of communication strategies were related to country of work, work environment, migration status, and migration and sex work stages. Participatory peer- based, workplace and m- health communication interventions could facilitate access to HIV/ STI prevention, and sexual and reproductive health and safety resources and information for migrant women involved in sex work, while strengthening peer support networks, social cohesion and community mobilization efforts.	8/10
Rhodes et al,^18^ 2020	North Carolin, United States	HOLA: a community-level, Spanish-language peer navigation Intervention to promote HIV testing and condom use	CBPR approach; Providing information; Support provision; HIV testing provision (referral)	RCT	Immigrant Spanish-speaking Latinx GBMSM and TW	The HOLA intervention is effective for increasing HIV testing among Latinx GBMSM and TW. At follow-up, HOLA participants reported increased HIV Knowledge (adjusted odds ratio = 2.2; 95% CI = 1.7–2.7; *P* < 0.0001) and increased HIV testing (adjusted odds ratio = 8.3; 95% CI = 3.0–23.0; *P*< 0.0001). In-depth interviews identified critical intervention elements and impacts and community needs and priorities.	9/13
den Daas et al,^40^ 2019	Netherlands	-	-	Qualitative	MSM with a non-western migration background	SNT-HIVST might overcome barriers to regular HIV testing including; being seen while testing, disclosure of sexual identity, and stigma related to HIV and sexual practices. Trust between the HIVST distributer and receiver was important. SNT-HIVST requires tailored peer support to address practical, informational, and emotional needs. MSM-NW distributing HIVST can have an important role in reducing health disparities in testing uptake among MSM-NW. Provided sufficient trust among MSM-NW; key factors found for successful implementation were education through an e-tool, and establishing quality support by a peer-coordinator for unanticipated questions.	7/10
Ryan et al,^41^ 2019	Melbourne, Australia	PRONTO!, as a peer-led RPOC HIV testing service, introduced free STI testing funded through Medicare (Australia’s universal healthcare system). Medicare ineligible migrant clients were required to pay for STI tests.	Community- based strategy; Providing information; HIV testing provision	Retrospective cohort study	Gay, bisexual and other MSM	STI testing uptake was significantly lower among Medicare ineligible clients (7.6%, 85.3%; *P* < 0.01). Following STI testing introduction there was an immediate increase in six-month return HIV testing (6.4%; *P* = 0.02) and a significantly increasing rate of return HIV testing between July 2016 and March 2018 (0.5% per month; *P*< 0.01) among Medicare eligible clients but no immediate change in return testing (0.9 %;* P* = 0.7) or the rate of change in return testing between July 2016 and March 2018 (0.1% per month; *P* = 0.3) among Medicare ineligible clients. In March 2018, six-month return HIV testing was 52.3% and 13.2% among Medicare eligible and ineligible clients respectively.	8/11
Febres-Cordero et al,^37^ 2018	Mexico-Guatemala border	-	-	Qualitative	Female sex workers	Peer support was found to be critical for reducing social isolation; improving access to HIV/STI knowledge, prevention and resources; and mitigating workplace violence, particularly at the initial stages of migration and sex work. Peer support was especially critical for countering social isolation, and peers represented a valuable source of HIV/STI prevention knowledge and resources (e.g., condoms), as well as essential safety supports in the workplace. Challenges to accessing peer support included difficulties establishing long-lasting relationships and other forms of social participation due to frequent mobility, as well as tensions among peers within some work environments. Variations in access to peer support related to country of work, work environment, sex work and migration stage, and sex work experience were also identified.	9/10
Khatoon et al,^38^ 2018	Nepal	-	-	Cross- sectional	Bhutanese refugees	The HIV testing and counselling services are utilized by less than a third (29%) of the key population among the Bhutanese Refugees. The prime source of information about the HIV testing and counselling sites has been health workers followed by peer/outreach educators and neighbors. Common self-reported barriers for utilization of HIV testing and counselling services by the Bhutanese refugees were self-perceived stigma about HIV, the fear of being discriminated and the lack of knowledge about HIV testing and counselling services.	5/8
Rosenberg and Bakomeza,^42^ 2017	Kampala, Uganda	To pilot a peer-education intervention tailored to meet the needs of refugee women engaged in sex work.	Community- based strategy; Providing information; Support provision; HIV testing provision	Interventional	Refugee women engaged in sex work	Findings from the pilot project suggest the feasibility of adapting existing rights-based and evidence-informed interventions with sex workers to humanitarian contexts. Findings further demonstrate how taking a community empowerment approach can facilitate these refugees’ access to a range of critical information, services and support options – from information on how to use contraceptives, to referrals for friendly HIV testing and treatment, to peer counselling and protective peer networks. As peer educators, participants expressed strong commitments to helping their peers access the same information and address the service gaps they have experienced. Respondents noted that peer-to-peer education is likely more effective than alternatives to outreach by, for example, public health actors, because they often have to work “in the shadows” and exercise discretion when discussing their experiences.	7/10
Alonzo et al,^30^ 2016	North Carolina, US	HOLA en Grupos: a small-group four-session Spanish language prevention intervention to increase condom use and HIV testing by Hispanic/ Latino MSM were delivered in Spanish by trained peer instructor/facilitators	CBPR approach; Providing information	RCT with qualitative analysis	Hispanic/Latino MSM and transgender persons	Six themes of intended behavior changes: increasing and maintaining condom use; identifying strategies to support correct and consistent condom use; increasing communication and negotiation with sexual partners about condom use; getting tested for HIV and other sexually transmitted infections; applying other sexual health promotion strategies; and sharing newly learned sexual health information with their peers.	7/10
Messengale et al,^32^ 2016	Central North Carolina, US	-	-	Qualitative	Latina immigrant women	Intrinsic enablers of HIV testing included individual trust, confidentiality, intergenerational family participation, and peers. The extrinsic enablers were local community outreach, bicultural/bilingual testing staff, service location and mass media outlets.	8/10
Tohme et al,^39^ 2016	Lebanon	-	-	Cross-sectional	Iraqi, Syrian, and Palestinian MSM refugees	According the bivariate model analysis, ever having been HIV tested was positively associated with having seen a doctor in the past year (82 vs 18 %; *P* < 0.001), being comfortable with medical doctors (58 vs 42 %; *P* < 0.05), knowing where to receive HIV testing (66 vs 44 %; *P* < 0.001), and spending time with other peer refugees (*P* < 0.01). In the multivariate models, The men who reported having seen a medical doctor in the previous year had 6 times greater odds of ever been HIV tested prior to the study (AOR = 5.98, CI 2.11, 16.96, *P* = 0.001). Participants who reported being comfortable with their doctor had greater odds of ever having been HIV tested (AOR = 2.47, 95 % CI 0.96, 6.38). The men who reported knowing where to find HIV testing had greater odds of ever having been HIV tested (AOR = 4.91, 95 % CI 1.90, 12.65, *P* < 0.001). Neither spending time with other refugees (AOR: 0.796; CI: 0.291, 2.235) nor experiencing discrimination based on their refugee status (AOR: 1.232; CI: 0.494, 3.073) were significantly associated with ever having been HIV tested.	7/8
Wagoner et al,^35^ 2015	North Carolina, US	A CBPR partnership developed, implemented, and evaluated the efficacy of an HIV/STD prevention intervention known as HoMBReS: (Men: Men Maintaining Wellbeing and Healthy Relationships), and used a LHA approach.^19^	CBPR approach; Providing information; Support provision; HIV testing provision (Referral)	Qualitative	Immigrant Latinos from soccer teams	Participants shared perceptions on social network importance for immigrant Latinos, facilitators and challenges of helping other men, recommendations for intervention modification and suggestions for future work involving the Latino community. Findings revealed that Latino men are receptive to fulfilling the roles of health advisors and opinion leaders, and can effectively serve as LHAs. Twelve themes emerged and were grouped into five domains: (i) context for helping among social networks of immigrant Latinos; (ii) Navegantes’ roles as LHAs; (iii) facilitators of helping; (iv) challenges of helping; and (v) recommendations from the LHAs. Social network members valued the social support they received. Working through sports teams and identifying existing leaders to be LHAs may be a culturally congruent approach to meeting Latino community needs.	7/10
Grieb et al,^31^ 2015	Baltimore, Maryland	-	-	Qualitative	Latino Immigrant Men	Four thematic categories emerged about the challenges and opportunities to accessing HIV testing and preventative services: information about HIV, HIV fear and stigma, barriers to accessing healthcare, and opportunities for intervention approaches. Information and communication technology provides an opportunity to improve access to HIV testing and prevention services. Individualized interventions, though, must be disseminated in collaboration with community-, structural-, and policy-level interventions that address HIV risk, HIV/AIDS stigma, and healthcare access among Latino immigrants.	7/10
Rhodes et al,^20^ 2011	North Carolina, US	HoMBReS-2 intervention: a small-group peer-led intervention designed to be interactive and activity-based. It included rapport and trusting building activities; didactic teaching; DVD segments that served as role modeling and triggers for discussion; role plays; group discussion; and skills building, practice, and feedback.	CBPR approach; Providing information; Support provision	RCT	Spanish-speaking, heterosexually active immigrant Latino men	HoMBReS-2 intervention was found efficacious in increasing HIV testing. Adjusting for baseline testing and covariates, intervention participants had higher HIV testing during the past 12 months than those in the comparison arm (AOR = 18.3; 95% CI = 3.59–92.9; *P* < 0.001). Again, examining sensitivity using multiple imputation (impute missing data), intervention participants had higher HIV testing during the past 12 months than those in the comparison arm, when adjusting for baseline HIV testing and covariates (AOR = 9.51; 95% CI = 3.52–25.6; *P* < 0.001).	8/13
Sena et al, 2010 ^33^	North Carolina, US	A community-based HIV testing strategy for Latino immigrants through door-to-door approach, which involves bringing HIV information and education to individuals directly in their homes through the use of promotores, who are trained Latino lay community health workers.	Community- based strategy; Providing information; HIV testing provision	Cross- sectional	Latino immigrant	Door-to-door rapid HIV testing is a feasible and acceptable strategy for screening high-risk Latino immigrants in the community. Nearly all participants who were surveyed supported community-based rapid HIV testing. The majority of them (n = 171) consented to rapid HIV testing, and 57 (25.0%) declined testing. The majority of participants (91.5%) preferred the rapid HIV test over the standard HIV testing method. Of those who preferred the rapid HIV testing method, 73.4% identified at home or in the community as their preferred location for receiving the test. In bivariate analysis, perceived HIV risk, no history of HIV testing, sex with a CSW, sex in exchange for drugs or money, living with a partner, and alcohol use were significantly associated with test acceptance. In the multivariate analysis, participants who had never been tested for HIV were more likely to consent to rapid HIV testing than those who had tested in the past (adjusted odds ratio 2.5; 95% CI, 1.1, 5.6). Most participants supported rapid HIV testing in the community (97%).	7/8
Ramos et al, 2009 ^17^	US-Mexico border	the Healthy Women project, as a six-month pilot study, placed promotoras in the unique role of animadoras who used a chain referral strategy called *Pasa la Voz* (Spread the Word) based on peer-driven intervention methodology	Community- based strategy; Providing information; HIV testing provision (Referral)	Interventional	Latino immigrant women	*Pasa la Voz* proved to be an effective strategy to reach Latinas at risk for HIV infection. The pilot study found that as a result of the Healthy Women project, Latinas at high to moderate risk of HIV infection became better informed about HIV prevention services in their community, accessed available services, and referred other at-risk Latinas from their social networks to HIV prevention services. It is significant that 53 (95%) of the seeds and all 61 (100%) individuals referred were tested for HIV. Initially, only 31 (55%) of the 56 seeds had any information about HIV prevention services in their area, but within six months, all 56 seeds (100%) had received information about HIV prevention services and had disseminated information about available services to at least 61 members of their social networks.	8/9
Rhodes et al, 2009 ^19^	central North Carolina, US	A CBPR partnership developed, implemented, and evaluated the efficacy of an HIV/STD prevention intervention known as HoMBReS: (Men: Men Maintaining Wellbeing and Healthy Relationships), and used a LHA approach.	CBPR approach; Providing information; Support provision; HIV testing provision (Referral)	Quasi-Experimental	Immigrant Latino men from Latino soccer teams	LHA interventions for Latino men that are developed in partnership with community members, rely on male centered intrapersonal networks, and are culturally congruent can enhance preventive behaviors and may reduce HIV infection. Relative to the control condition, participants in the intervention were more likely to report HIV testing (AOR = 2.5; CI = 1.5-4.3, *P* = 0.001). Working within the naturally existing social network of a rural soccer league, Navegantes were able to increase HIV testing, knowledge of HIV transmission and prevention among their teammates.	8/9
Vissman et al, 2009 ^34^	North Carolina, US	*HoMBReS : Hombres Manteniendo Bienestar y Relaciones*Saludables (Men: Men Maintaining Well-being and Healthy Relationships) was an LHA intervention to reduce HIV and STD risk and increase use of HIV and STD health care services among recently arrived, non-English-speaking Latino men who were members of a multicounty Latino soccer league in central North Carolina.	CBPR approach; Providing information; Support provision; HIV testing provision (Referral)	Qualitative	Immigrant Latino men who were members of a multicounty soccer league	*Navegantes*described the function and facilitators of serving as LHAs and identified leverage points for future HIV and STD prevention strategies. They highlighted psychosocial and sociocultural influences on HIV risk, settings for risky behavior, and personal changes from serving as *Navegantes*. This study provides preliminary evidence that an LHA approach is feasible and appropriate for Latino men, and can be effective in reaching men who might otherwise be difficult to reach.	8/10

Abbreviations: QA: quality appraisal; GBMSM: Gay, bisexual and other men who have sex with men; TW: transgender women; SNT-HIVST: social network testing with hiv self-tests; CBPR: community based participatory research; HoMBReS: Hombres Manteniendo Bienestary Relaciones Saludables; LHA: Lay Health Adviser; STD: sexually transmitted disease; RPOC: rapid point-of-care; CSW: commercial sex worker.

###  Results of studies’ quality appraisal

 Regarding the criteria (particularly relevance) for the quality assessment of the studies, all the studies included data that researchers had used to test the initial program theory. In addition, a total of 84 CMOCs were extracted from these 17 studies, indicating the appropriate quality of such studies in terms of relevance. Moreover, the qualitative evaluation results also revealed that most of these studies (n = 15) obtained the necessary criteria (over 70%) in the qualitative appraisal tool. On the other hand, the lowest number of CMOCs was extracted from the remaining two studies (see Table 2). Besides, the required explanations are given in the results section for the pathways.

###  Concepts of program theory

####  1. Intervention/service strategies

 All the 10 interventional studies (Studies in which an intervention or a service was provided) employed multiple strategies. The strategies were categorized into the following four groups:

####  1.1. Providing information about HIV and available services

 This strategy has been used in 10 interventional studies.^17-20,30,33-35,41,42^ It is crucial to note that the design and implementation of interventions/services were highlighted according to the characteristics, needs, and conditions of immigrants and provided the necessary information accordingly, particularly based on the Community-Based Participatory Research (CBPR) approach.^18-20,30,34,35^ These studies have used multiple methods or materials to provide information, including group discussions based on immigrants’ culture and language, as well as sharing information through PowerPoint, DVDs, role-playing scenarios, group activities, or the distribution of brochures. The training was provided individually or in groups. In addition, the peers would have undergone training before peer intervention/service provision.

####  1.2. Providing support

 This strategy has been used in 6 studies,^18-20,34,35,42^ where peers acted as supporters or advocators for the immigrants and provided formal and informal assistance to immigrants to meet their needs and overcome existing obstacles during the service/intervention provision. These supports included providing information and counseling, contacting the members of the social network, accompanying the immigrants to take the test, defending the immigrants while facing violence, etc.

####  1.3. Community-based strategy

 This strategy has been used in all 10 interventional studies. Six studies have used the CBPR approach and attempted to increase awareness and change the attitude and behavior of immigrants through community participation.^18-20,30,34,35^ Moreover, the peers in other studies have also implemented community-based strategies to provide outreach and chain referral services,^17^ door-to-door outreach services,^33^ outreach educational training, support and referral services,^42^ and STI/HIV testing services directed by the peers in a national community-based study.^41^

####  1.4. Provision of HIV testing services

 Providing HIV testing services was identified as one of the strategies. Two studies indicated that rapid HIV testing was performed by the intervention team (including peers).^33,41^ However, in other studies, peers only suggested the immigrants take HIV tests and referred them to the center.

####  2. Contextual factors

 Influential contextualfactors were categorized into three groups of individual/interpersonal factors (language, values, and beliefs, socio-economic status, immigration status, fear and stigma, gender), health service-related factors (accessibility, availability, acceptability, affordability), and structural factors (regulations and policies, supportive resources, discrimination).

####  3. Mechanisms and pathways of peer interventions for HIV testing uptake

**Figure 3:**
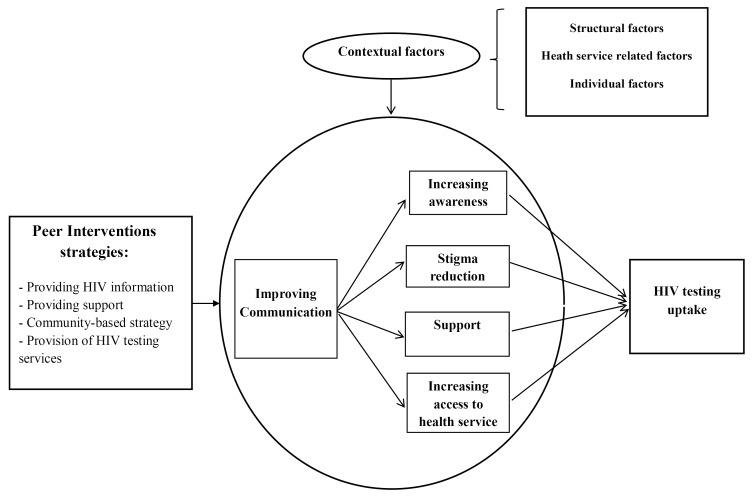
The refined program theory explaining the effect of peer interventions on immigrants’ HIV testing uptake

 According to the review results, four mechanisms that were proposed in the initial program theory, including increasing awareness, reducing stigma, supporting, and increasing access to services, were confirmed. In addition, the improvement of communication was identified as a proximal mechanism. Peers’ familiarity with social network members, their ability to reach more immigrants, and their ability to build trust could play a significant role in developing effective communication and generating other mechanisms. After comparing the existing mechanisms in all the 84 CMOCs extracted, 4 pathways were identified. Eventually, the refined program theory resulted from the realist review (Figure 3).

###  Pathway 1. Following improved communication, increasing awareness could lead to improved HIV testing uptake

 This pathway was identified according to 37 CMOCs extracted from 12 studies.^17-19,30,33-38,40,42^ Besides, 8 studies were interventional (intervention/service delivery), using multiple strategies to perform the intervention.^17-19,30,33-35,42^ Familiarity with and trust in peers as well as the comfortable feeling while talking about sex with peers (i.e., improved peer communication with immigrants in the target group), could facilitate the knowledge exchange. Peers can help immigrants use HIV testing services by sharing knowledge, correcting misunderstandings, as well as increasing immigrants’ awareness of HIV and available services. According to the qualitative evaluation results regarding this pathway, 11 studies reported acceptable quality (score: ≥ 70%). One study with a score of < 70%,^38^ accounts for only two of the 37 extracted CMOCs. Therefore, in total, the evidence related to this pathway can be considered of acceptable quality.

###  Pathway 2. Following improved communication, the reduction of stigma could lead to improved HIV testing uptake

 This pathway was identified according to the evidence from 8 CMOCs, which were extracted from 5 studies.^17,18,33,34,40^ In 4 studies, the intervention/service was provided.^17,18,33,34^

 Some studies have reported that the peers’ performance characteristics has led to an increase in the use of tests among immigrants, including building trust and also creating an enabling environment where immigrants experience less stigma (by providing outreach, door-to-door, and rapid testing services, as well as ensuring the confidentiality of the information).^18,33^ The fear of stigma was regarded as a critical factor leading to the failure of the intervention/service program. Immigrants would avoid interacting with peers due to stigma issues and fear of disclosing information to their family members,^17^ or lack of trust and fear of rejection by peers;^34^ thus, they would not perform HIV testing despite peer support. The evidence pertaining to this pathway was reported to have acceptable quality. The qualitative evaluation of these studies revealed acceptable scores ( ≥ 70%) for these 5 studies.

###  Pathway 3. Following improved communication, enhanced support would lead to lead to improved HIV testing uptake

 The third pathway was identified according to the evidence from 17 CMOCs, which were extracted from 7 studies.^18,20,35-37,40,42^ 4 studies were conducted based on the provision of intervention/service.^18,20,35,42^ Peers can effectively facilitate successful implementation of interventions and improvement of using HIV tests among immigrants by means of developing communications within trust through information support, empathy, encouraging immigrants to demonstrate health behaviors, accompanying them while taking HIV test services, and expressing a strong commitment to providing continuous support to these immigrants. The qualitative evaluation results revealed that six studies reported an acceptable quality (over 70%). Notably, only one of the 17 cases of CMOCs related to this pathway was extracted from one study with a quality appraisal score of < 70%.^20^

###  Pathway 4. Following improved communication, increasing access to services would lead to improved HIV testing uptake

 This pathway was identified according to the evidence from 22 CMOCs, which were extracted from 11 studies.^17-19,32-34,36,37,39-41^ 6 studies reported provision of intervention/service using multiple strategies.^17-19,33,34,41^ Following effective communication, there was an improvement in taking HIV tests among immigrants. Such an enhancement was the result of different interventions, which were performed through mechanisms related to improving access to services (e.g., the specialization of activities in accordance with the immigrants’ needs and different priorities in their social network, providing acceptable services to this group, providing various services such as rapid and accessible HIV testing, providing outreach HIV testing services during non-working hours, improving peers’ access to a large number of members of the target community).^18,19,34^ While financial support and access to free testing services could play a critical role in immigrants’ HIV testing uptake, one study reported that the high cost of testing, as well as the limitation of using private insurance, led to a reduction in taking HIV testing among immigrants.^41^ Contextual factors, such as lack of continuity of service delivery, inaccessible services, or perceived long waiting times, have been regarded as crucial challenges for some immigrants.^17^ The evidence pertaining to this pathway revealed an acceptable quality, where the findings of the qualitative appraisal reported an obtained score of over 70% for these 11 studies.

## Discussion

 In order to achieve the first target of the *95-95-95 HIV testing and treatment targets* (95% of people within the subpopulations who are living with HIV know their HIV status), it is imperative to design and implement interventions aiming to improve HIV testing in immigrants as one of target sub-populations.^5^ This realist review revealed how a successful peer intervention under certain contextual circumstances might trigger mechanisms to improve HIV testing uptake among international immigrants. These interventions encompassed multiple strategies and were implemented in accordance with immigrants’ needs and conditions as well as the existing HIV testing barriers.

 According the results, the interventions aimed to improve the use of HIV testing by raising awareness of HIV, reducing stigma, supporting, and increasing access to testing services. The “increasing HIV awareness” mechanism had the highest frequency among the reviewed studies in the first pathway, which was the result of 37 cases of CMOCs extracted from 12 studies. Accordingly, immigrants as peers were able to play an important role in increasing immigrants’ HIV knowledge through access to a large number of social network members, creating effective communication and building trust to talk and share knowledge about sexual and HIV issues, and moderate misunderstandings. Linguistic skills and cultural knowledge of peers could also facilitate the establishment of effective communication and knowledge transfer by decoding and interpreting the consequences of using available services. Among various community-based educational models, peer education models are recognized as an effective approach to raising awareness as well as access to HIV/AIDS services, specifically for hard-to-reach populations.^13,14^

 The results showed that peer interventions through the stigma reduction mechanism could increase HIV testing in this group. The number of CMOCs related to this pathway was lower and was extracted from only 5 studies. Accordingly, these interventions helped improve the use of HIV testing in immigrants by reducing perceived or experienced stigma as a result of creating a safe environment, reducing the fear of experiencing stigma, implementing community-based strategies with confidentiality considerations, as well as providing outreach and door-to-door services. In particular, it was facilitated by peers’ skills and capabilities to build trust for talking about sexual issues that were considered taboo in society. A meta-synthesis study also revealed that the easy acceptance of each other in the peer support worker teams created a safe and positive work environment, reduced stigma, and increased recovery.^43^ Sharing experiences and speaking a common language as peers might lead to the building of trust, the involvement of these populations in related interventions, and the use healthcare services.^44^

 The results further showed that providing services by peers through support could lead to increased HIV testing in immigrants. Peer support was typically provided in the form of assistance, accompanying immigrants to HIV testing centers, or knowledge sharing. In the meantime, establishing effective communication through trust-building prepares the ground for efficient social support. In addition, other factors, including the acquisition of communication skills as well as the ability to understand the contextual conditions and needs of the target group, could play an essential role in providing appropriate support to peers. It was assumed that peers, who are similar to the target group in terms of social identity, experiences, and social role, are more likely to support the target group in a way that even non-peer professionals might be unable to perform. It is also noteworthy that the power difference between the service recipient and the provider (as a peer) would be minimized in the case of peer support interventions. It can help and motivate service recipients, especially marginalized groups who are reluctant due to fear of stigma and discrimination, to involve in the intervention.^12^

 Moreover, the findings indicated that the interventions attempting to improve various dimensions of access to services, including the provision of accessible, available, affordable, as well as acceptable services in line with the immigrants’ needs, attitudes, and priorities, led to desirable results concerning the rate of HIV testing among immigrants. Conversely, HIV testing would be less common among immigrants due to different restrictions, such as lack of financial or geographical access to such services. The peers’ personal and functional characteristics were assumed to play a crucial role in the acceptance of services and the increase in HIV testing in immigrants. Similarly, peers’ access to a large number of community members and their ability to reach out to distant individuals, particularly based on chain referral strategies, could lead to increased referrals and testing by a greater population of immigrants. Evidence has also confirmed the value of peer support models in improving access to such services in target groups.^43^ These findings highlight the need to pay attention to different aspects of access to services while designing and implementing interventions. Consequently, these services should be accessible, available, continuous, affordable, and in line with the target group’s culture and attitude, so HIV testing will be promoted in the immigrant group.

 According to the refined program theory, the process of implementation of interventions and the decision-making by immigrants on whether to use these available services and opportunities are affected by several contextual factors, including individual/interpersonal factors, health service-related factors, and structural factors. Interventions that effectively address such barriers could succeed in improving HIV testing among immigrants. In contrast, such intervention may not be fully effective if immigrants continue to encounter barriers while accessing HIV testing services. These findings emphasize the necessity to consider various contextual factors at micro, meso, and macro levels while designing interventions to increase HIV testing and diagnosis and reduce disease transmission. These findings confirm previous evidence regarding the contextual factors influencing access to HIV services. In this regard, previous systematic review studies have highlighted a wide range of factors affecting access to HIV services at various levels.^10,45^

 It is also noteworthy to mention that most studies were conducted in the United States (n = 12), where the major focus is on the Latin American immigrant population. According to the reports of IOM, Asia and Europe hosted 84 million and 82 million international immigrants in 2019, respectively. These figures represent 62% of the world’s total population of international immigrants. North America accounts for 22 percent of the world’s immigrant population, followed by Africa (10%), Latin America and the Caribbean (4%), and Oceania (3%).^46^ It is also likely that some interventions have been conducted or are being implemented, but there have been no reports in the literature. These findings are consistent with the results of another systematic review study on the interventions aiming to increase the use of HIV testing in immigrants. Since the researchers asserted that almost all of the related interventional studies were conducted in the United States,^47^ therefore, the finding of the present realist review may indicate the necessity for further research in different countries with a focus on immigrant populations.

## Strengths and limitations

 This study can be regarded to identify the strategies, mechanisms, and contextual factors of interventions to improve using HIV testing among international immigrants. The refined theory extracted from the present realist review can be used as a framework for policymakers and researchers to design interventions aimed at increasing HIV testing with respect to contextual factors. Considering the heterogeneity of the international immigrant population in terms of individual characteristics and especially different social, economical, political, and cultural structures in different countries, the findings cannot be generalized to a specific context. Therefore, researchers and policy makers should use the findings of the present study by considering the context in which their intervention or program is to be developed, implemented and evaluated. The researchers were also faced with another limitation, including the selection of studies that targeted only international immigrants, which would lead to the exclusion of interventions on a broader range of groups such as domestic immigrants, non-immigrant ethnic/racial minorities, or indigenous populations. Therefore, it is suggested to consider a wider variety of populations for future studies.

## Conclusion

 In immigrants population, as a vulnerable group in HIV control programs, peer interventions with multiple strategies to be designed and implemented considering the barriers to HIV testing and also moving beyond one-size-fits-all approaches can successfully improve HIV testing uptake. Employing peers as an available resource in the community is effective for designing and implementing interventions based on the immigrants’ culture and needs. By addressing contextual factors, peer interventions for immigrants could develop effective communication, raise awareness of HIV, reduce stigma, improve support, and increase access to HIV services. As a comprehensive framework, the presented theory can help policymakers and decision-makers in HIV/AIDS control programs promote the use of HIV testing in immigrants and reduce the risk of disease transmission.

## Acknowledgements

 The authors are grateful for the financial support of Tehran University of Medical Sciences (TUMS), Grant No: 96-03-62-36567.

## Competing Interests

 The authors declare that they have no competing interests.

## Ethical Approval

 Not applicable.
